# Single-Crystal Structural Analysis of 2D Metal–Organic Frameworks and Covalent Organic Frameworks by Three-Dimensional Electron Diffraction

**DOI:** 10.1021/acs.accounts.4c00335

**Published:** 2024-08-15

**Authors:** Qichen Chen, Guojun Zhou, Zhehao Huang

**Affiliations:** † Center for Electron Microscopy, School of Emergent Soft Matter, South China University of Technology, Guangzhou, Guangdong 510640, China; ‡ Department of Materials and Environmental Chemistry, Stockholm University, Stockholm SE-106 91, Sweden

## Abstract

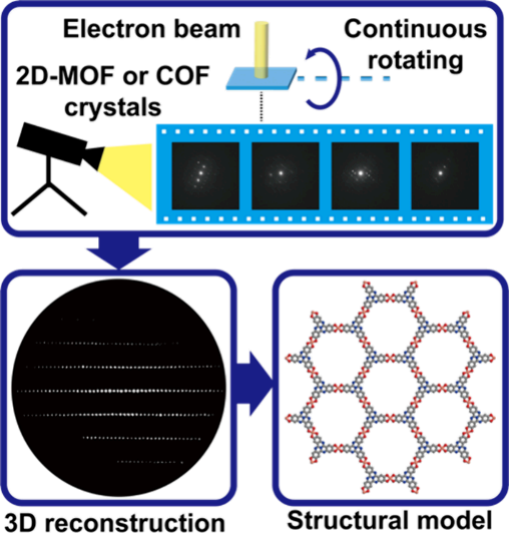

In the development of 2D metal–organic frameworks (MOFs) and 2D covalent organic frameworks (COFs), obtaining structural details at the atomic level is crucial to understanding their properties and related mechanisms in potential applications. However, since 2D-MOFs and COFs are composed of layered structures and often exhibit sheet-like morphologies, it is challenging to grow large crystals suitable for single-crystal X-ray diffraction (SCXRD). Therefore, *ab initio* structure determination, which refers to solving the structure directly from experimental data without using any prior knowledge or computational input, is extremely rare for 2D-MOFs and COFs. In contrast to SCXRD, three-dimensional electron diffraction (3DED) only requires crystals sized in tens or hundreds of nanometers, making it an ideal method for single-crystal analysis of 2D-MOFs and COFs and obtaining their fine structural details.

In this Account, we describe our recent development of the 3DED method and its application in structure determination and property studies of 2D-MOFs and COFs. A key development is the establishment of a continuous 3DED data collection protocol. By collecting electron diffraction (ED) patterns continuously while performing crystal tilting, the electron dose applied to the target nanocrystal is greatly reduced. This allows the acquisition of high-resolution 3DED data from 2D-MOFs and COFs by minimizing their damage under the electron beam. We have also developed an approach to couple 3DED with real-space structure solution methods, i.e., simulated annealing (SA), for single-crystal structural analysis of materials that do not have high crystallinity. We successfully determined two 2D-COF structures by combining 3DED with SA.

We provide several examples demonstrating the application of 3DED for the *ab initio* structure determination of 2D-MOFs and COFs, revealing not only their in-plane structures but also their stacking modes at the atomic level. Notably, the obtained structural details serve as the foundation for further understanding the properties of 2D-MOFs and COFs, such as their electronic band structures, charge mobilities, etc. Beyond structure determination, we describe our work on using 3DED as a high-throughput method for the discovery of new materials. Using this approach, we discovered a novel MOF that was present only in trace amounts within a multiphasic mixture. Through this discovery, we were able to tune the synthesis conditions to obtain its pure phase.

We detail how 3DED can be used to probe different levels of molecular motions in MOFs through the analysis of anisotropic displacement parameters (ADPs). Additionally, we show that 3DED can provide accurate information about intermolecular weak interactions such as hydrogen bonding and van der Waals (vdW) interactions. Our studies demonstrate that 3DED is a valuable method for the structural analysis of 2D-MOFs and COFs. We envision that 3DED can accelerate research in these fields by providing unambiguous structural models at the atomic level.

## Key References

- ZhouG.; YangT.; HuangZ.Structure Determination of a Low-Crystallinity Covalent Organic Framework by Three-Dimensional Electron Diffraction. Commun. Chem.2023, 6, 116.37286771 10.1038/s42004-023-00915-4PMC10247803.^1^ This work demonstrates that the structures of COF nanocrystals can be solved from low-resolution data (1.5) Å by combining 3DED with simulated annealing. The resulting structural model is similar to that obtained from high-resolution data (0.9 Å) by the dual-space method.
- GeM.; WangY.; CarraroF.; LiangW.; RoostaeiniaM.; SiahrostamiS.; ProserpioD. M.; DoonanC.; FalcaroP.; ZhengH.; ZouX.; HuangZ.High-Throughput Electron Diffraction Reveals a Hidden Novel Metal–Organic Framework for Electrocatalysis. Angew. Chem. Int. Ed.2021, 60 ( (20), ), 11391–1139710.1002/anie.202016882PMC825258633682282.^2^ By investigating a large number of nanocrystals, a new zeolitic imidazolate framework (ZIF) is discovered in a trace amount among another known ZIF material. This demonstrates 3DED can be used as a high-throughput method for discovering new materials.
- KangC.; YangK.; ZhangZ.; UsadiA. K.; CalabroD. C.; BaughL. S.; WangY.; JiangJ.; ZouX.; HuangZ.; ZhaoD.Growing Single Crystals of Two-Dimensional Covalent Organic Frameworks Enabled by Intermediate Tracing Study. Nat. Commun.2022, 13, 137035296677 10.1038/s41467-022-29086-xPMC8927472.^3^ This is the first high-resolution (0.9 Å) 2D-COF structure obtained *ab initio* by 3DED. In addition to the AA stacking model, an unprecedented six-layer stacking behavior is observed.
- SporrerL.; ZhouG.; WangM.; BalosV.; RevueltaS.; JastrzembskiK.; LöfflerM.; PetkovP.; HeineT.; KucA.; CánovasE.; HuangZ.; FengX.; DongR.Near IR Bandgap Semiconducting 2D Conjugated Metal–Organic Framework with Rhombic Lattice and High Mobility. Angew. Chem. Int. Ed.2023, 62, e20230018610.1002/anie.20230018636862366.^4^ The structure of the 2D-MOF is determined by 3DED. It presents a rare slipped AA stacking model at the atomic level. This demonstrates the advantages of using 3DED to obtain detailed layer stacking information from 2D-MOFs.
- ZhangJ.; ZhouG.; UnH.-I.; ZhengF.; JastrzembskiK.; WangM.; GuoQ.; MückeD.; QiH.; LuY.; WangZ.; LiangY.; LöfflerM.; KaiserU.; FrauenheimT.; Mateo-AlonsoA.; HuangZ.; SirringhausH.; FengX.; DongR.Wavy Two-Dimensional Conjugated Metal–Organic Framework with Metallic Charge Transport. J. Am. Chem. Soc.2023, 145 ( (43), ), 23630–2363837852932 10.1021/jacs.3c07682.^5^ 3DED reveals the unique 2D structure with wavy layers. The wavy layers in this 2D-MOF affect its surface charge distribution and electronic band structure.

## 1 Introduction

Metal–organic frameworks (MOFs) and covalent organic frameworks (COFs) represent two classes of porous materials, which have attracted intense research interest in recent decades. The frameworks of MOFs are commonly connected between metal-oxo clusters or cations with organic linkers through coordination bonding,^6,7^ whereas those of COFs are connected through strong covalent bonds between organic monomers.^8^ Reticular chemistry has been a cornerstone in the design of MOFs and COFs at the molecular level,^9^ expanding their applications in gas capture and storage,^10−14^ separations,^15−18^ catalysis,^19−21^ sensing,^22,23^ biomedical applications,^24−26^ etc.

While the fields of MOFs and COFs have been undergoing rapid development, 2D materials have shown unique physical and chemical properties that are unattainable by their 3D counterparts.^27^ The rise of 2D-MOFs and COFs has brought versatile properties, leading to their potential applications in various fields.^28−32^ However, the crystallization process of 2D-MOFs and COFs often produces crystals with sheet-like morphologies. As the shortest length of one dimension is in the range of nanometers, it poses a great challenge for structural analysis by single-crystal X-ray diffraction (SCXRD), which, in turn, hinders our understanding of the underlying structure–property relationships. Although powder X-ray diffraction (PXRD) has been used for the structure determination of 2D-MOFs and COFs, peak overlap can prevent the distinction between different structural models.

In the light of such challenges, three-dimensional electron diffraction (3DED) has emerged as an important single-crystal analysis method since 2007.^33−36^ It utilizes transmission electron microscopy (TEM) for data acquisition. Compared to X-ray, the strong interaction between electrons and matter enables it to provide high signal-to-noise ratio data from nanocrystals. Recently, the progress on collecting 3DED data continuously while tilting the target nanocrystal has significantly reduced the applied electron dose on the samples.^37−42^ As a consequence, it allows *ab initio* structure determination to directly obtain structural models of MOFs and COFs from experimental data, while the compounds can be easily damaged under electron beam.^43−45^

In this Account, we detail our efforts toward single-crystal structure determination of 2D-MOFs and COFs. We describe the development of data collection and structure solutions focusing on MOFs and COFs as the analytes. We use several 2D-MOFs and COFs as examples to highlight the significance of obtaining structural information at the atomic level and their impact for related research . In addition, we describe the uniqueness of applying 3DED to discover novel materials and study physicochemical properties such as molecular motions and weak intermolecular interactions. Furthermore, the challenges and opportunities of the 3DED method are discussed.

## 2 3DED Method

**Figure 1:**
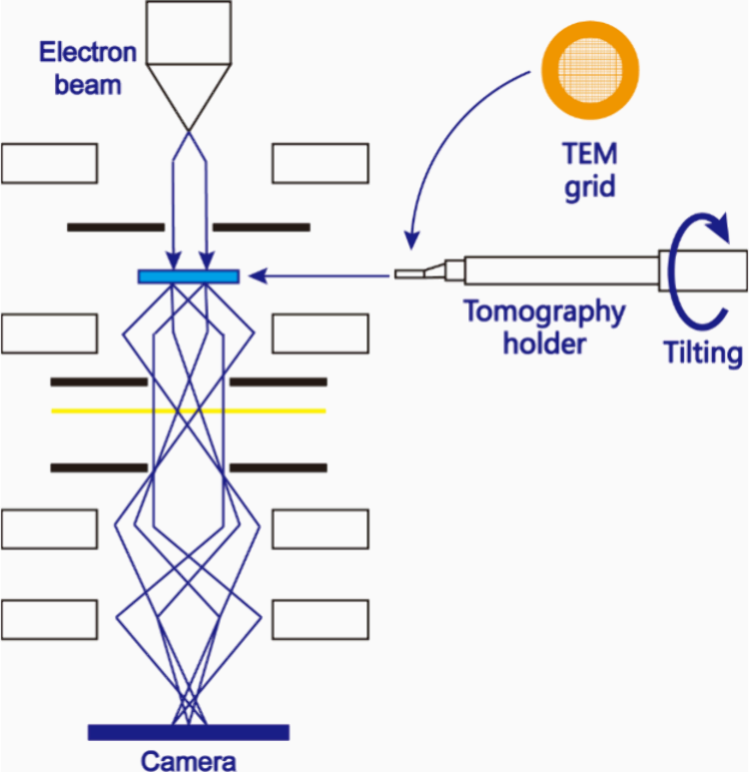
Illustration of the instrumentation of 3DED.

**Figure 2:**
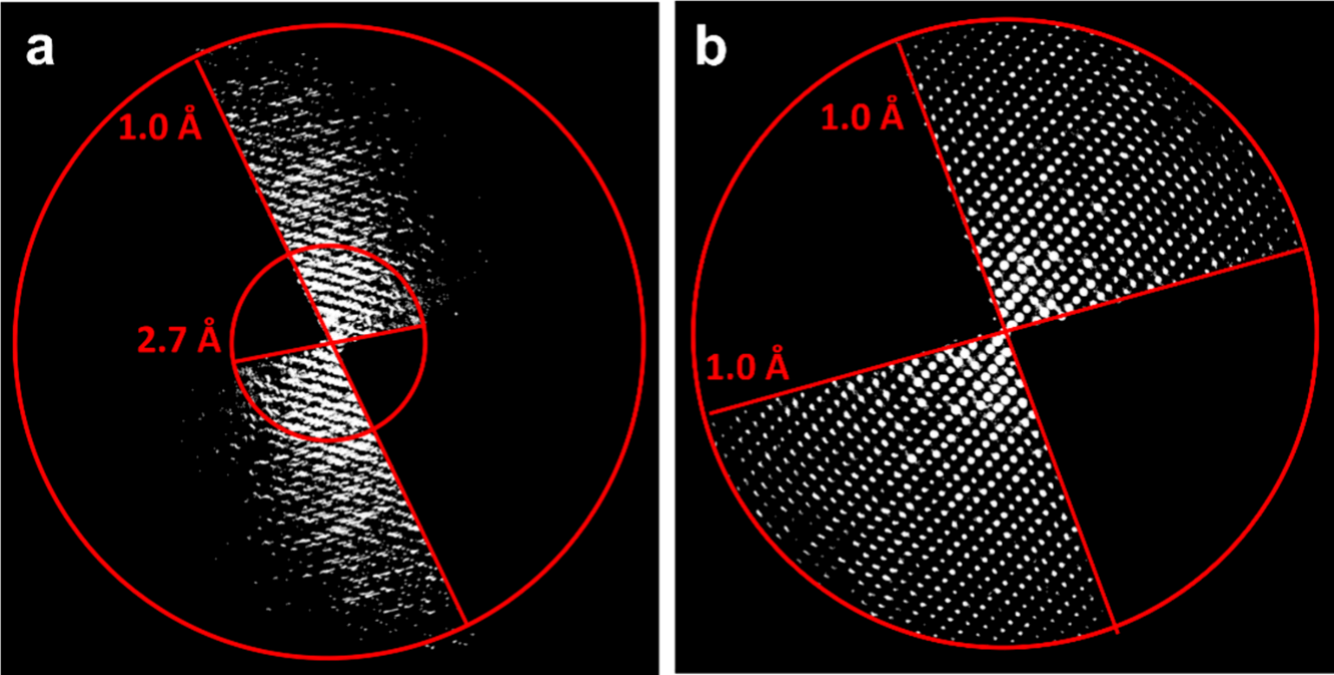
Reconstructed 3D reciprocal lattice of PCN-226 from 3DED data collected by (a) stepwise rotation and (b) continuous rotation.

Electrons behave differently from X-rays when they interact with matter. Electrons are charged particles, while an X-ray is electromagnetic wave. In addition, the common wavelength of electrons produced in a TEM, e.g., 0.02508 Å for 200 kV TEMs and 0.01969 Å for 300 kV TEMs, is much shorter than X-rays, e.g., 0.7107 Å from the Mo source. The resulting strong interaction between electrons and matter underpins the use of TEMs to obtain a high signal-to-noise ratio for nanocrystals for their single-crystal analysis. The development of 3DED started with several stepwise methods for collecting 3DED data.^46,47^ Typically, nanocrystals are spread on a TEM grid. The TEM grid is further loaded in a tomography holder. With the rotation of the TEM goniometer, an electron diffraction (ED) pattern can be obtained at each rotation angle, achieving fine sampling of 3D reciprocal space (Figure 1). Although data completeness is limited by the goniometer rotation range, compared to studying structures using ED data taken from main-zone axes, this advancement significantly improved data coverage, simplified data collection, and reduced dynamical effects. The stepwise method have found huge success in analyzing inorganic materials such as zeolites^48^ and intermetallics.^49^ However, studies of MOFs and COFs using the stepwise method are rare^50,51^ because it often finds that the sample crystallinity decreases rapidly during data collection, as indicated by the decreasing data resolution (Figure 2a). This results from the nature of hybrid materials and organic crystals that makes them more sensitive to electron beam damage, *i.e.*, radiolysis, compared to inorganic crystals.

To tackle this challenge, we developed a continuous rotation electron diffraction (cRED) data collection protocol with Zou and co-workers.^37,38^ Instead of collecting an ED pattern at each rotation angle, the continuous method records a series of ED patterns while the goniometer is rotating continuously. As a result, the data collection process is ∼20× faster than stepwise methods. Hence, this method greatly reduces the accumulated electron dose applied to the crystal, minimizes beam damage, and allows the acquisition of high-resolution data from MOFs and COFs (Figure 2b). In addition, the continuous method yields integrated diffraction intensities, which are more accurate than those obtained by stepwise rotation. More practical details and troubleshooting can be found in our recently published protocol.^36^ Furthermore, it is worth noting that several different groups have independently developed different protocols for collecting data continuously, *e.g.*, Fast-EDT,^40^ Fast-ADT,^41^ MicroED,^52^ and cPEDT,^53^ while sharing the same core concept.

**Figure 3:**
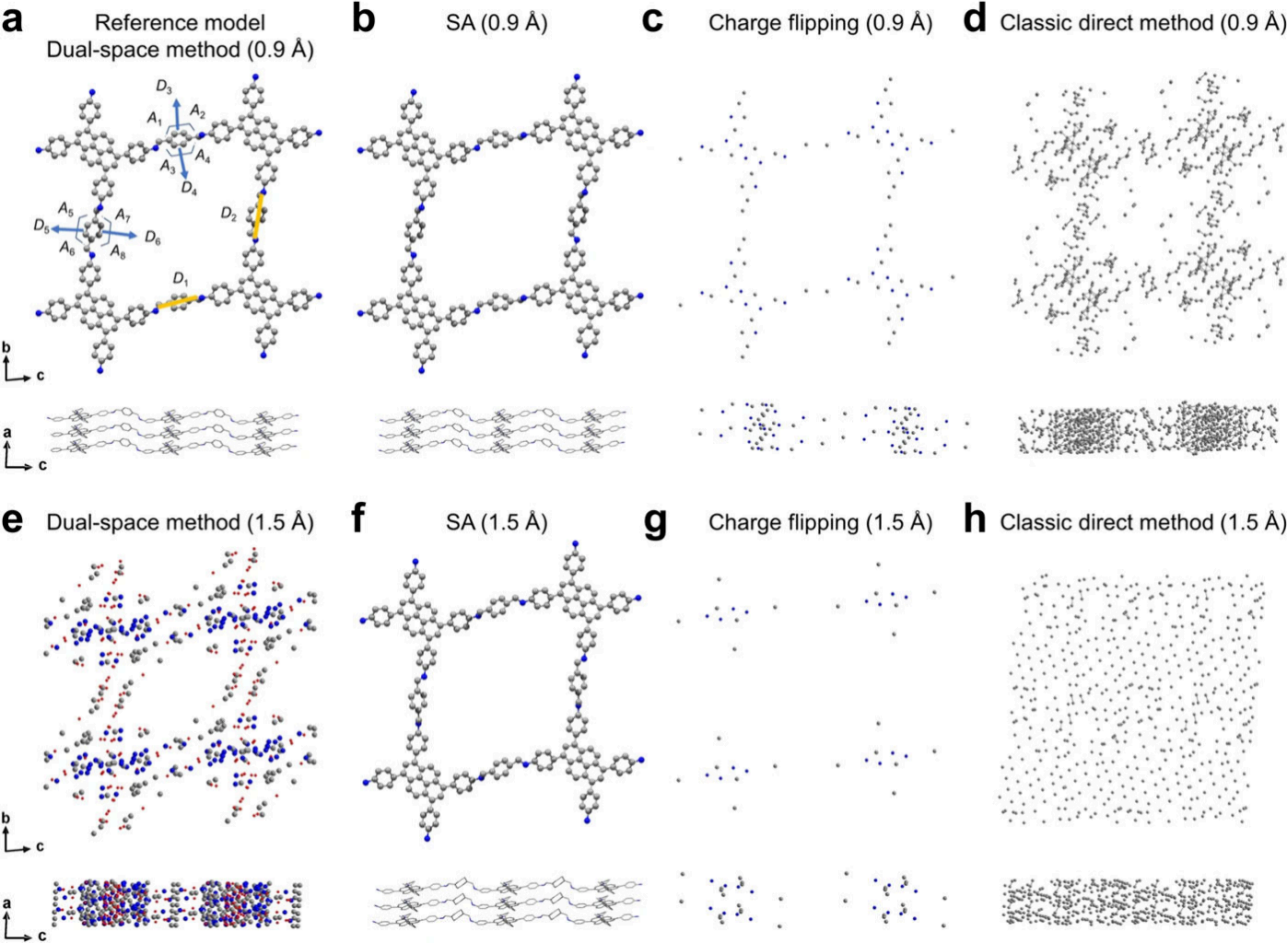
Structural models of Py-1P created using 0.9 Å resolution 3DED data obtained by (a) the dual-space method, (b) the SA method, (c) charge flipping, and (d) the classic direct method. The structural models of Py-1P created using 1.5 Å resolution 3DED data obtained by (e) the dual-space method, (f) the SA method, (g) charge flipping, and (h) the classic direct method. Reprinted with permission from ref (1). Copyright 2023 Springer Nature.

As the continuous method shares a similar data acquisition strategy with SCXRD, it enables us to use X-ray crystallography software for structure determination. Structure determination of MOFs and COFs usually involves using XDS^54^ for data processing, the dual space method implemented in SHELXT^55^ for structure solution, and SHELXL^56^ for least-squares refinement. However, 2D-MOFs and, especially, 2D-COFs are often challenging to crystallize in high crystallinities. As the data resolution is limited, it could prevent unambiguous structure determination. We demonstrated that low-resolution 3DED data, coupled with simulated annealing (SA), can provide the model of a 2D-COF, Py-1P, as good as that obtained from high-resolution data.^1^ Because SA is a real-space structure solution method, it can take advantage of our chemical knowledge about the building units for the synthesis of MOFs and COFs. We found that for a 3DED data resolution as low as 1.5 Å, SA was able to obtain a model similar to that obtained from 0.9 Å data resolution, while other structure solution methods such as the dual-space method, the classic direct method, and charge flipping failed to produce a chemically meaningful model (Figure 3).

**Figure 4:**
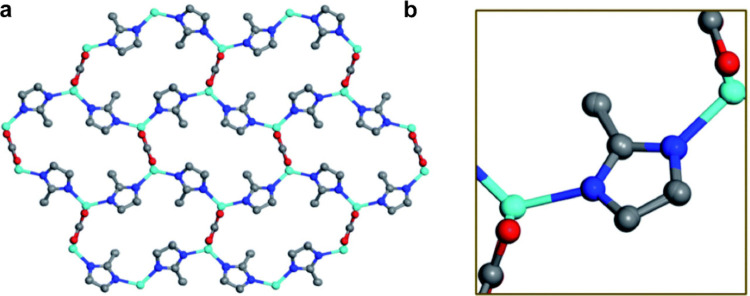
(a) The structural models obtained from 3DED and SCXRD are superimposed and viewed along the *c*-axis. The heavy atom, Zn, deviates by 0.06(1) Å, and the light atoms deviate by 0.07(3) Å on average. The positional difference of each atom is too small to be observed in the superimposed structures, showing the good agreement of both methods. (b) A zoomed-in area showing the mIM linker and Zn(II) cations, showing the largest positional difference on the C atom from the methyl group. Blue spheres, N; red spheres, O; gray spheres, C; cyan spheres, Zn atoms. Reprinted with permission from ref (57). Copyright 2021 the Royal Society of Chemistry.

While the strong interaction between electrons and matter brings us the advantage of a high signal-to-noise ratio, it also leads to multiple scattering. As a result, 3DED data typically have a larger *R*_1_ value compared to that from SCXRD data. We therefore investigated the accuracy of 3DED by comparing the results for a MOF, ZIF-CO_3_-1, obtained by 3DED and synchrotron SCXRD.^57^ We found that the obtained unit cell parameters are very close, and the largest difference was 0.65% for the *b*-parameter. We further compared the structural models. The single heavy atom, Zn, deviates by 0.06(1) Å, whereas the light atoms have an average positional deviation of 0.07(3) Å. The bond lengths and angles show average deviations of 0.04(3) Å and 4(3)°, respectively. This indicates that despite the high *R*_1_ values from 3DED data, the structural models derived from 3DED and SCXRD show a high degree of consistency (Figure 4). In addition, unlike using a kinematical assumption to calculate structure factors, dynamical refinement^58^ can be used to compare dynamical data and dynamical calculated structure factors.

## 3 Structural Analysis of 2D-MOFs and COFs

**Figure 5:**
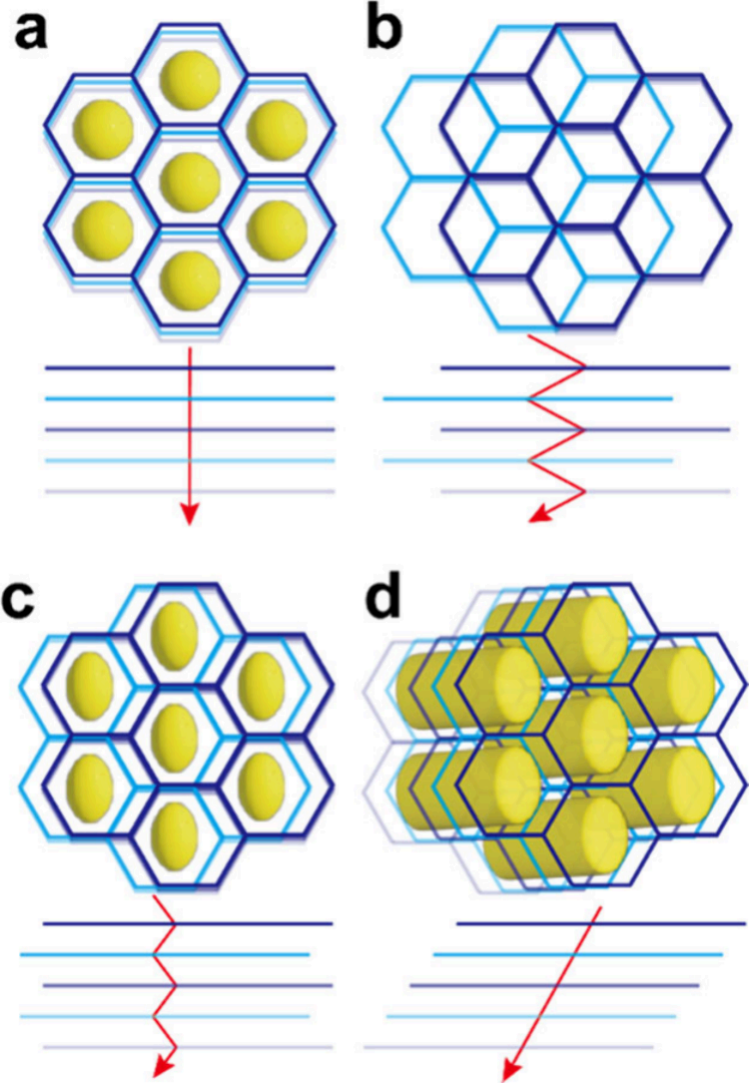
Illustration of (a) eclipsed stacking (AA stacking), (b) staggered stacking (AB stacking), (c) inclining stacking (inclined AA stacking), and (d) serrated stacking (slipped AA stacking).

The determination of 2D-MOF and COF structures is crucial for gaining insights into their structure–property relationships. As 2D materials, the structures of 2D-MOFs and COFs can be divided into two parts: the in-plane structures and interlayer stacking behaviors. It is obvious that the in-plane structure has a decisive influence on the properties of the materials through the selection of metals and the functionalities of the organic linkers. On the other hand, interlayer stacking also plays an important role in defining porosity, electronic and photonic properties, etc.^59^ Until recently, the most reported stacking modes were eclipsed stacking, staggered stacking, inclining stacking, and serrated stacking^28,59,60^ (Figure 5). However, due to the short dimension challenge for SCXRD, *ab initio* structure determination of 2D-MOFs and COFs had hardly been achieved. Therefore, to analyze the structure, several structural models are usually proposed first according to chemical knowledge. Then, computational studies combined with different characterization methods, such as high-resolution transmission electron microscopy (HRTEM) imaging, PXRD, N_2_ sorption, X-ray absorption spectroscopy (XAS), etc., are used to validate the proposed models.

**Figure 6:**
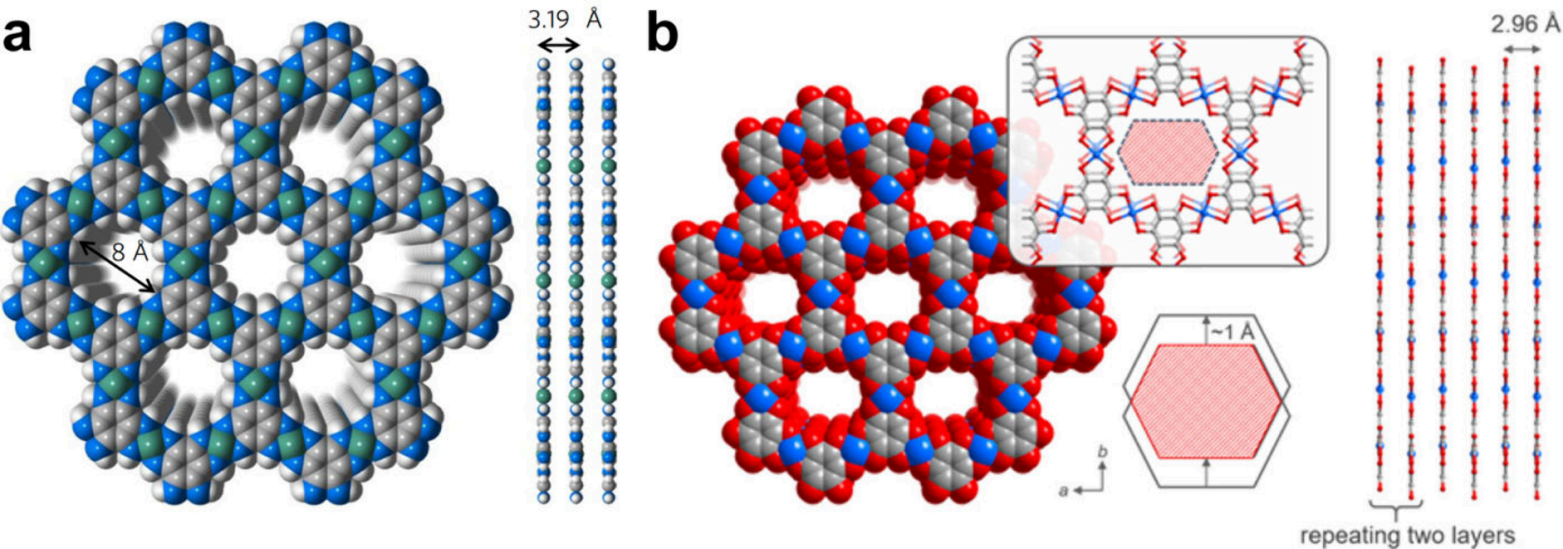
Structural models of (a) Cu-HAB and (b) Cu-HHB. Reprinted with permission from refs (32) and (61), respectively. Copyright 2018 Springer Nature and 2018 American Chemical Society, respectively.

For example, we investigated the structure of a 2D conductive MOF, Cu-HAB (HAB = hexaaminobenzene), which performs excellently for enhancing energy storage in supercapacitors.^32^ We first obtained high-resolution synchrotron PXRD data. However, Pawley fitting using eclipsed and staggered models yields similar results due to peak broadening and overlapping in the PXRD patterns. Meanwhile, HRTEM imaging, selected area electron diffraction (SAED), and N_2_ sorption are able to distinguish the porosity and structural differences. Combined with computational studies, we confirmed that Cu-HAB has an eclipsed structure (Figure 6a). We also investigated the structure of another 2D-MOF, Cu-HHB (HHB = hexahydroxybenzene). From the PXRD pattern, we observed a shoulder peak at ∼4.6° (λ = 0.45236 Å). This is a feature that can distinguish the serrated Cu-HHB model from its eclipsed counterpart. Nevertheless, we cannot rule out that the shoulder peak can also be attributed to other factors such as impurities. Thus, we reach the conclusion by combining the PXRD analysis with observation results from HRTEM imaging, which showed elliptical pores, and density functional theory (DFT) calculations (Figure 6b).^61^

## 4 Single-Crystal Analysis of 2D-MOFs and COFs by 3DED

In the wake of such complications, we started to explore *ab initio* structure determination of 2D-MOFs and COFs. The development of a continuous 3DED protocol has advanced the field of single-crystal analysis of nanosized crystals under low electron dose conditions, which are essential for 2D-MOFs and COFs. It is worth mentioning that as a single-crystal structural analysis method, 3DED requires a thickness larger than 20 nm to obtain high-quality Bragg reflections along the layer directions.

**Figure 7:**
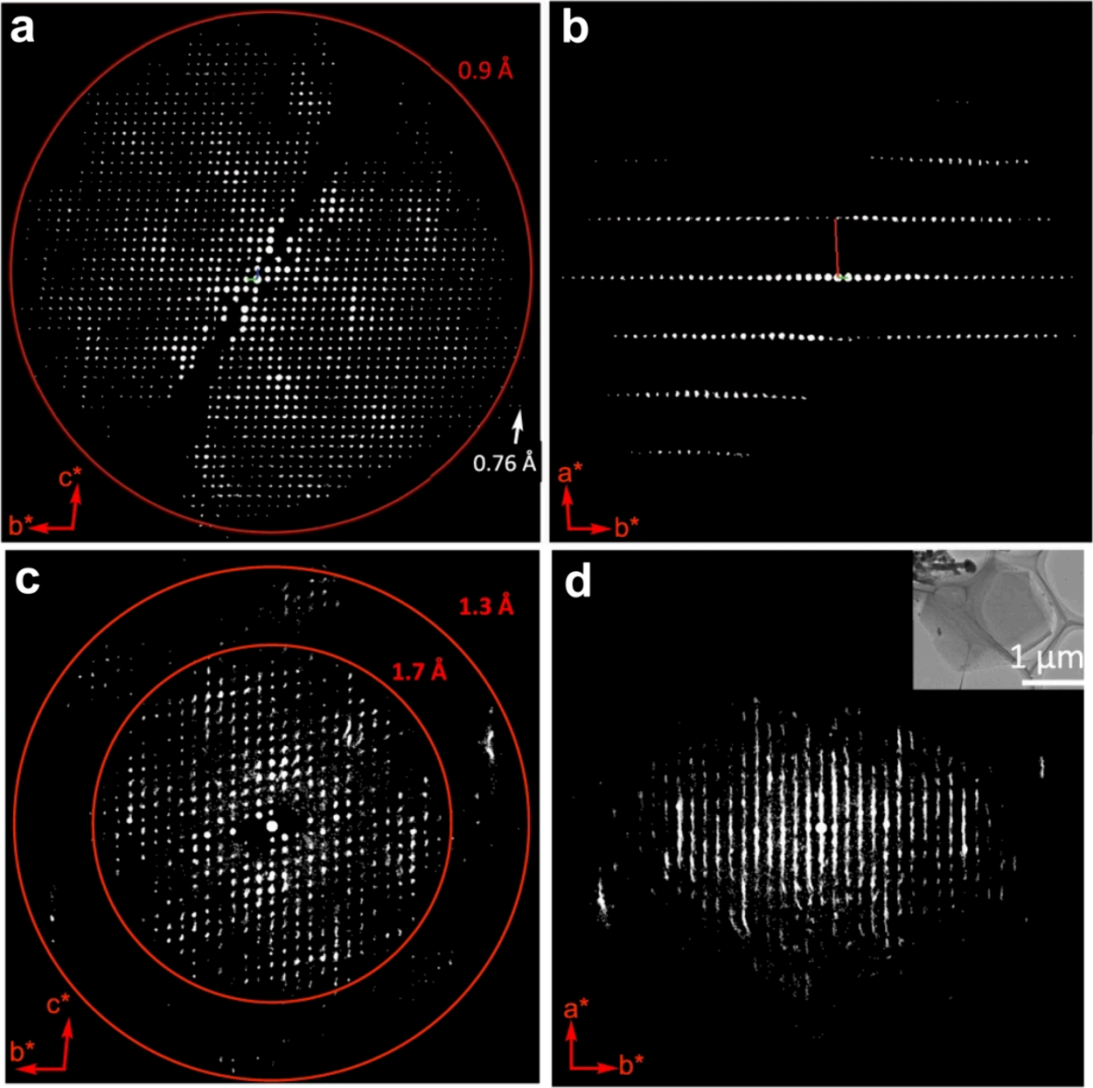
Reconstructed 3D reciprocal lattice of Py-1P from the 3DED data viewing along the (a, c) [100] and (b, d) [010] directions. A different a*-axis shown in parts b and d indicated the different stacking behavior. Reprinted with permission from ref (3). Copyright 2022 Springer Nature.

We reported the first high-resolution single-crystal structure of a 2D-COF, namely, Py-1P constructed by 4,4′,4″,4‴-(1,9-dihydropyrene-1,3,6,8-tetrayl)-tetraaniline (DTA) and (1,4-phenylene)bis(*N*-phenylmethanimine) (PPA).^3^ The obtained 3DED data had a high signal-to-noise ratio within the resolution of 0.90 Å and the highest diffraction observed at a resolution of 0.76 Å. *Ab initio* structure solution by the dual-space method implemented in SHELXT can directly locate 58 C and N atoms among the total number of 60. It reveals the AA stacking structure of Py-1P, with an interlayer distance of 3.7 Å. Because 3DED provides data that can be reconstructed in 3D reciprocal space, it is especially powerful for distinguishing different stacking behaviors in 2D-MOFs and COFs. For example, AA stacking usually has an interlayer spacing of 3.2–3.7 Å, which corresponds to the 0.27–0.31 Å^–1^ distance between diffraction planes in reciprocal space. AB stacking has the same interlayer spacing of 3.2–3.7 Å but contains two layers in one period. Therefore, it corresponds to the 0.14–0.17 Å^–1^ distance between diffraction planes. This provides us an unambiguous way to distinguish AA and AB stacking. In the study of Py-1P, the in-plane structures are similar, as indicated by the similar intensity distributions from the reflections in the b*c*-plane (Figure 7a and c). Meanwhile, different stacking behaviors can be observed along the a*-axis (Figure 7b and d). Although it contains a certain degree of disorder, we discovered a rare six-layer stacking behavior. Calculating from the high-resolution region, the *a*-parameter is about six times that to form the AA stacking structure, indicating six layers stack along the *a*-axis to form the periodicity (Figure 7d).

**Figure 8:**
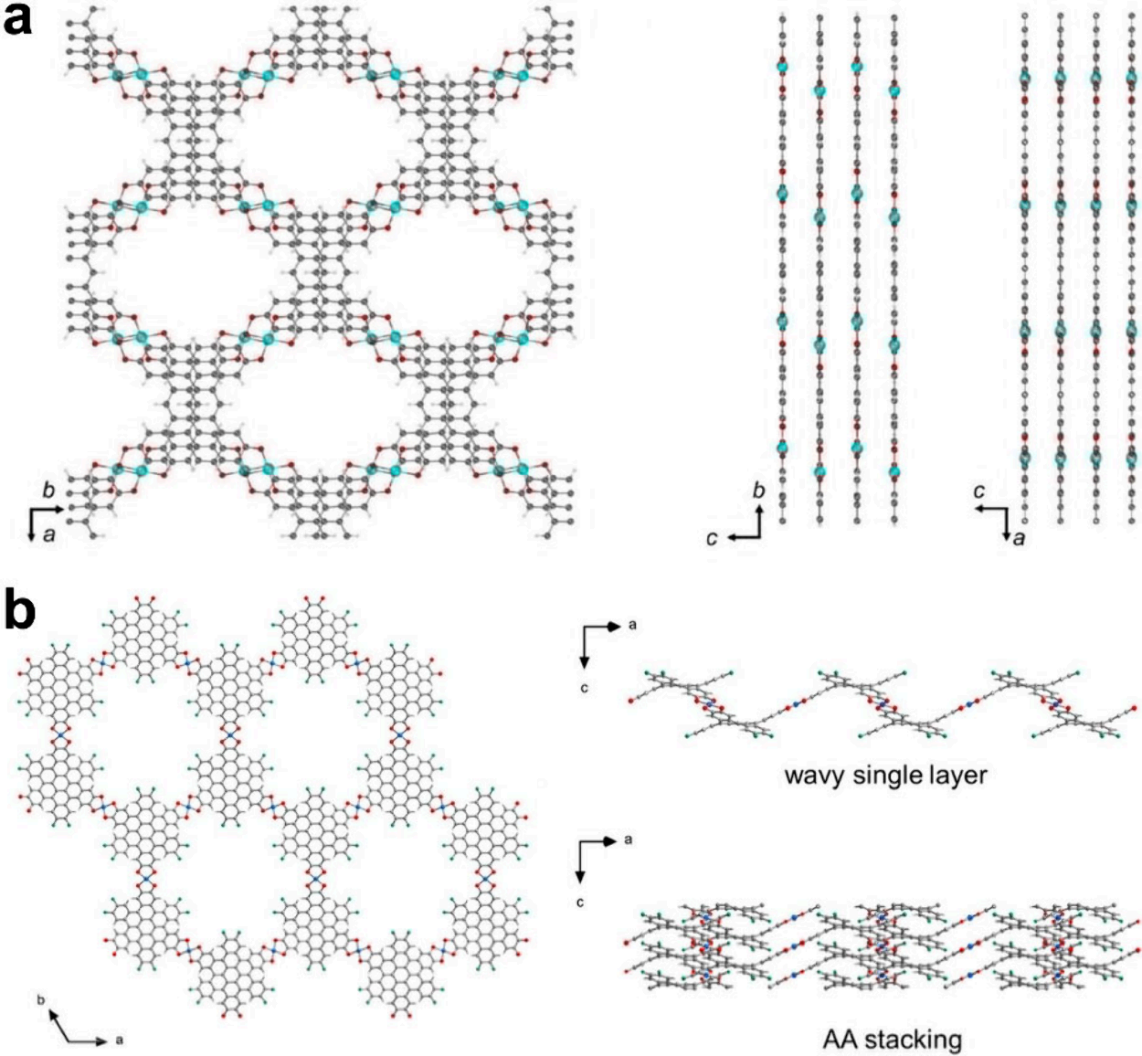
(a) The structural model of Cu_2_(OHPTP) showing a slipped AA stacking structure with a *ca*. 2 Å layer shift along the *b*-axis. (b) The structural model of Cu_3_(HFcHBC)_2_ featuring the wavy layers. Reprinted with permission from refs (4) and (5), respectively. Copyright 2023 Wiley-VCH GmbH and 2023 American Chemical Society, respectively.

2D-MOFs features characteristic unconventional electronic properties, yet their structures have been determined *ab initio* only in rare occasions.^62^ We studied the structure of a 2D-MOF, Cu_2_(OHPTP) (OHPTP = 2,3,6,7,11,12,15,16-octahydroxyphenanthro[9,10:*b*] triphenylene), by using 3DED. The 3DED data reached a high resolution of 0.90 Å. The obtained unit cell parameters (*a* = 22.74 Å, *b* = 21.58 Å, and *c* = 6.50 Å) indicated a possible AB stacking structure. After processing the 3DED data, we were able to determine the structure *ab initio*, showing that Cu_2_(OHPTP) has a rare slipped AA stacking structure (Figure 8a).^4^ More importantly, single-crystal analysis reveals the ∼2 Å layer shift along the *b*-axis. The quantitative analysis of the layer offset could be difficult for other characterization methods. Knowing the detailed structural features, we further conducted computational studies using the resolved structural model, which shed light on the mechanism of the high charge mobilities of Cu_2_(OHPTP).

Due to reaction kinetics and thermodynamics, not all 2D-MOFs and COFs can have high crystallinity, which leads to high 3DED data resolution. However, single-crystal analysis by 3DED can still provide crucial structural information. For example, in the study of Cu_3_(HFcHBC)_2_ (HFcHBC = 2,3,10,11,18,19-hexafluoro-6,7,14,15,22,23-hexahydroxy), the obtained 3DED data have a resolution of 1.8 Å. The unit cell parameters (*a* = *b* = 27.719 Å, and *c* = 3.93 Å) reveal a possible AA stacking structure. Although the 3DED data resolution was not sufficient for direct methods and the dual-space method, we obtained the structural model by combining 3DED data and the SA method. The result indicates an AA-eclipsed stacking model having an in-plane honeycomb lattice and wavy layers (Figure 8b).^5^ Cu_3_(HFcHBC)_2_ exhibited a metallic nature with a conductivity of 5.2 S cm^–1^ at room temperature. Based on the previously unseen wavy structure, we further studied the surface charge distribution and electronic band structure of Cu_3_(HFcHBC)_2_, providing insights into its metallic behavior.

## 5 3DED for Novel Material Discovery and Property Studies

**Figure 9:**
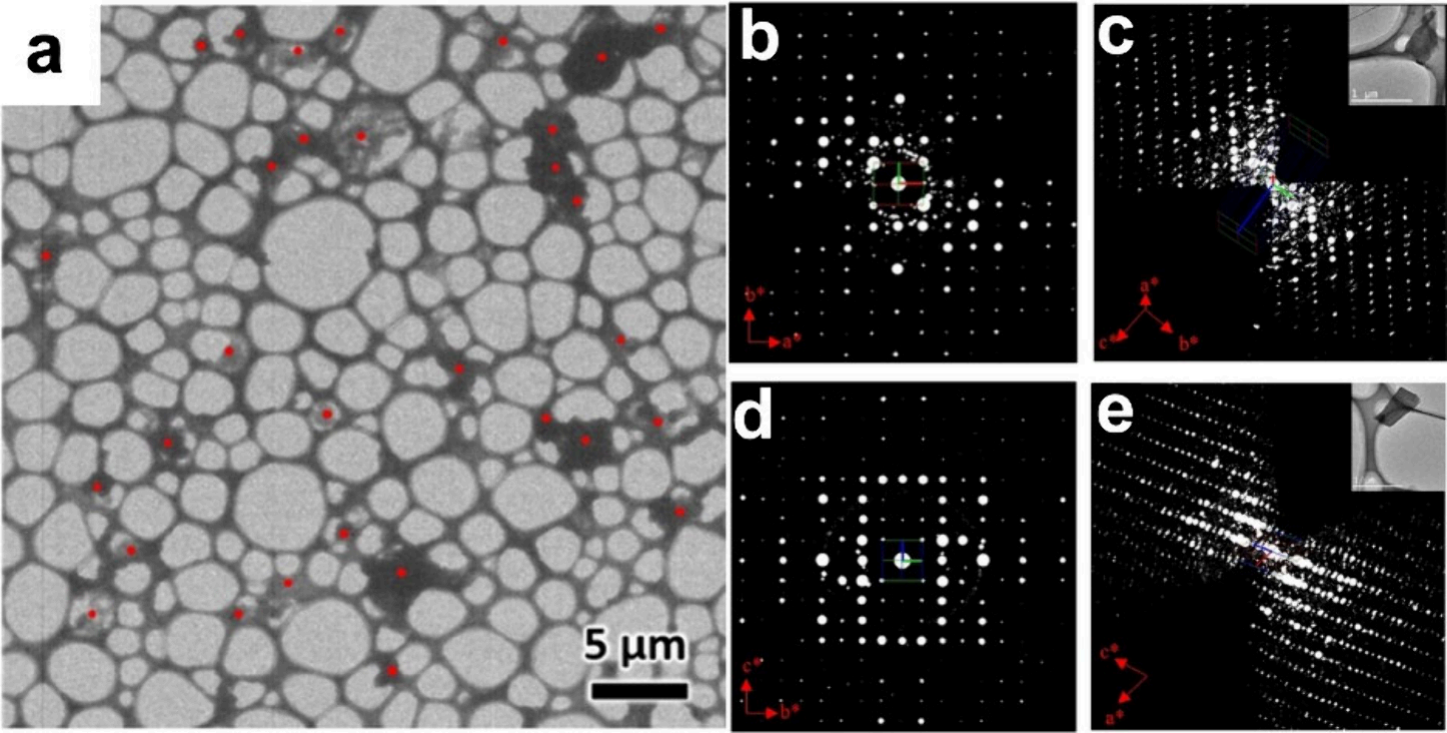
(a) TEM image showing individual nanocrystals (marked by red dots) in an area of 35 × 35 μm^2^ studied by 3DED. The 3D reconstructed reciprocal lattice of (b and c) ZIF-CO_3_-1 and the newly discovered (d and e) ZIF-EC1. Reprinted with permission from ref (2). Copyright 2021 Wiley-VCH GmbH.

In the synthesis of 2D-MOFs and COFs, it is common to obtain multiphasic polycrystalline powders as the product. While these are challenging to study by X-ray diffraction, 3DED offers a unique opportunity to study each nanocrystal. Moreover, due to the establishment of a continuous 3DED protocol, it now takes 3–5 min to collect 3DED data from a nanocrystal. This allows for the high-throughput analysis of individual nanocrystals, enabling the determination of each structure within a phase mixture. In our recent study, we have demonstrated the use of 3DED for the discovery of a novel MOF, ZIF-EC1, which is present in a trace amount in the large quantity of the phase mixture.^2^ The discovery was made after analyzing more than 30 nanocrystals in an area of 35 × 35 μm^2^, and structure determination by 3DED showed two crystals have the novel structure of ZIF-EC1 (Figure 9). Meanwhile, with the identification of each phase in the mixture, the overlapped diffraction peaks in PXRD can then be indexed and understood. Furthermore, by knowing the structure of ZIF-EC1, we were able to optimize the synthesis conditions and finally obtained the pure ZIF-EC1 product. Because ZIF-EC1 has high Zn and N contents and the bridging O atom can lead to mesopores after pyrolysis, we found the converted carbon material was an excellent electrocatalyst for the oxygen reduction reaction.

**Figure 10:**
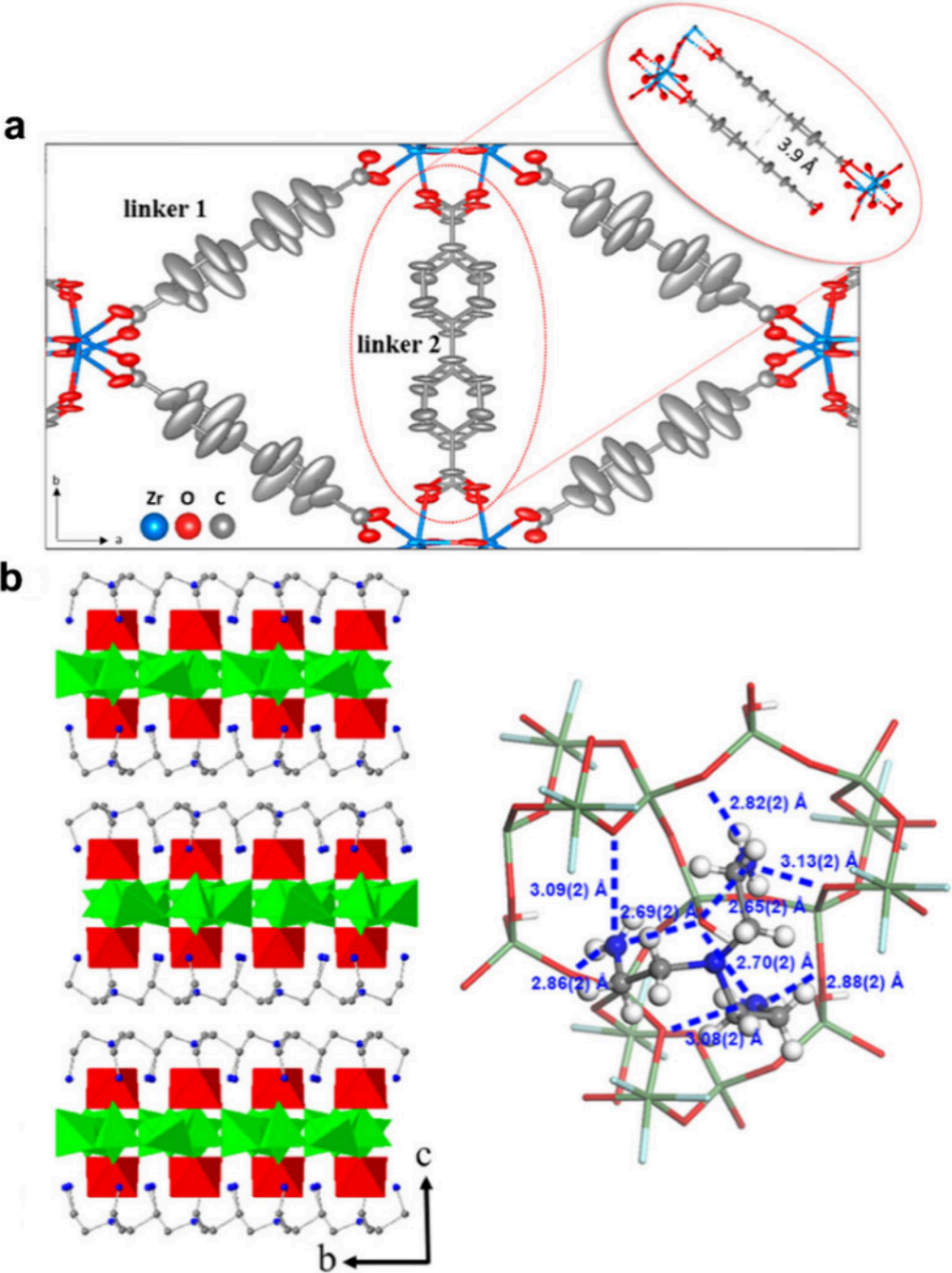
(a) Thermal ellipsoid model (50% probability) of MIL-140C obtained after refinement against 3DED data, showing different levels of motion in linkers 1 and 2. (b) The layered structural model of SU-68 and the H-bond network between TAEA molecules and the framework. Reprinted with permission from refs (63) and (64), respectively. Copyright 2021 and 2022 American Chemical Society, respectively.

3DED also provides the capability to study properties that could be difficult to characterize by other methods. By analyzing anisotropic displacement parameters (ADPs) obtained from 3DED data, we highlight the potential by using 3DED to probe molecular motions within MOF and COF nanocrystals.^63^ We studied UiO-67 and MIL-140C under both room and cryogenic temperatures to identify the molecular motions. The small-amplitude librations in the 4,4′-biphenyldicarboxylate (bpdc) linker can be indicated from the thermal ellipsoid models of UiO-67 and MIL-140C, where the librating C atoms show large and elongated ADPs. Furthermore, by analyzing ADPs from the same bpdc molecule at different positions in MIL-140C, we are able to differentiate the various degrees of molecular motions of the same linker (Figure 10a). Utilizing an ultralow electron dose rate, we have proved that 3DED can be used to study weak intermolecular interactions.^64^ SU-68 is constructed by 2D porous GeO_2_ layers, and the layers are connected through intermolecular weak interactions among tris(2-aminoethyl)-amine (TAEA) molecules. Using 3DED, we identified H-bonding between TAEA and the GeO_2_ layer, along with van der Waals (vdW) interactions between TAEA molecules (Figure 10b). Further analysis of the H-bond length revealed the relative bond strength among the different H-bonding sites.

## 6 Summary and Outlook

This Account summarizes our contributions toward understanding structural details of 2D-MOFs and COFs. 3DED has been developed as a powerful single-crystal analysis method. With the recent development on continuous data collection, electron beam damage can be minimized and thus facilitate the acquisition of high-resolution 3DED data from 2D-MOFs and COFs. By revealing the detailed structures of 2D-MOFs and COFs, it allows us to gain a deep understanding of the structure–property relationships of these materials. In the extension of obtaining structural details, 3DED has shown great potential in the discovery of new materials, as well as the study of unique structural properties, such as probing different extents of molecular motions and identifying weak intermolecular interactions. With commercialized and dedicated electron diffractometers already on the horizon, we believe 3DED will become more and more accessible to general research groups, increasing its importance in accelerating research in the fields of 2D-MOFs and COFs.

However, development of 3DED is still in its infancy, and many challenges and opportunities remain in this field. For example, current refinement of MOF and COF structures mainly uses a kinematical assumption, *e.g.*, as implemented in SHELXL,^56^ yet dynamical effects lead to ED intensities that deviate from the kinematical ones.^65^ Developing dynamical refinement of 2D-MOFs and COFs could provide additional structural insights. Moreover, while we have coupled 3DED with SA, further development is needed to study more complex MOF and COF structures with low-resolution data. Currently, training a nonexpert to collect and process 3DED data would require a time frame from months to years. Although progress has been made on automation of the processes, much effort is required to develop 3DED methods toward full automation. On the other hand, while SCXRD has established standards for assessing data quality, result accuracy, and other metrics over several decades, 3DED has yet to establish similar standards that nonexperts can use as a reference. Establishing such standards would require the collective effort of the community. Overall, we hope this Account offers insight into 3DED and its potential as a vital single crystal analysis method for 2D-MOFs and COFs.
